# Reporting Diarrhoea through a Vernacular Term in Quechua-speaking Settings of Rural Bolivia

**DOI:** 10.3329/jhpn.v29i6.9890

**Published:** 2011-12

**Authors:** Gonzalo Durán Pacheco, Andri Christen, Ben Arnold, Jan Hattendorf, John M. Colford, Thomas A. Smith, Daniel Mäusezahl

**Affiliations:** ^1^Department of Epidemiology and Public Health, Swiss Tropical and Public Health Institute, Basel, Switzerland; ^2^University of Basel, Basel, Switzerland; ^3^School of Public Health, University of California-Berkeley, CA, USA

**Keywords:** Definitions, Diarrhoea, *K'echalera*, Perceptions, Bolivia

## Abstract

Field studies often use caregiver-reported diarrhoea and related symptoms to measure child morbidity. There are various vernacular terms to define diarrhoea that vary across the local cultural contexts. The relationship between vernacular definitions of diarrhoea and symptoms-based definitions is not well-documented. This paper describes the association of the vernacular Quechua term *k'echalera* with the symptoms-based standard definition of diarrhoea in rural Bolivian settings. During a cluster randomized trial in rural Bolivia, both signs and symptoms of diarrhoea and reports of *k'echalera* were collected for children aged less than five years. Reported *k'echalera* were found to be associated with important changes in stool frequency, consistency, and presence of blood and mucus. Reported *k'echalera* were highly related to three of four recorded categories of watery stool. The intermediate (milk-rice) stool consistency, which fits into the definition of watery stool, was not strongly related to *k'echalera*. Mucus in the stool was also associated with *k'echalera*; however, its presence in *k'echalera*-free days accounted for at least 50% of the possible false negatives. The sensitivity and specificity of the term *k'echalera* were estimated by Bayesian methods, allowing for both symptoms of diarrhoea and reports of *k'echalera* to be subject to diagnosis error. An average specificity of at least 97% and the sensitivity of at least 50% were obtained. The findings suggest that the use of *k'echalera* would identify fewer cases of diarrhoea than a symptom-based definition in rural Bolivia.

## INTRODUCTION

Based on a common set of signs and symptoms, diarrhoea is defined as the obvious change in the normal stool pattern, characterized by three or more watery loose stools in a 24-hour period, or one or more stools with evident presence of blood or mucus (1-4).

Reports of mothers or caregivers are also used and widely accepted for reporting the occurrence of diarrhoea in children (5-7). Vernacular terms must then be employed, and morbidity estimates may be calculated from these. The validity of such reports is supported by the observation that people who regularly care for young children are aware of the actual change in the child's normal habits of stool frequency, volume, and consistency (1,3). The correspondence between mother-defined and symptom-based definitions may vary across populations and cultures (1).

A generic term *k'echalera* is widely used in Quechua-speaking settings of South America (from northern Ecuador to southern Bolivia). It refers to a change in the ordinary stool patterns as a result of an increased volume and frequency of stool with simultaneous change of stool consistency. The term has also been adopted as part of the folk and *Criollo* language in urban Spanish-speaking areas in Bolivia (8) and is used by health and medical staff to assess diarrhoea in rural areas. Eleven specific terms (e.g. *k'echa pukay*, *k'echa k'ellu*, *k'echa yuraj*) have been found to classify gastrointestinal illness by colour, odour, and frequency of stool, with *k'echalera* representing a general term for watery and frequent stool (9).

It is widely recognized that cultural definitions of diarrhoea may not correspond perfectly to symptom-based definitions that are often used in epidemiologic studies (10). This paper aimed at assessing the meaning of this vernacular term compared to the symptoms-based standard definition, thereby identifying the differences between the cultural and the conceptual understanding of diarrhoea. We also estimated the sensitivity and specificity of the vernacular definition compared to the international standard.

## MATERIALS AND METHODS

### Data

We used data from a baseline survey and the first six months of the post-intervention follow-up of a recent community randomized trial on solar water disinfection. The trial was conducted in rural Bolivia during October 2004−June 2006. The baseline survey included 819 children aged less than five years, and 725 children were followed up after randomization (11). Information on weekly and daily diarrhoeal symptoms and occurrence of *k'echalera* were collected for the eight-week baseline and the post-intervention follow-up respectively. Mothers or primary caregivers of the study participants provided information on the number of stools during the last 24 hours, stool consistency, presence of blood or mucus, and occurrence of *k'echalera*. In focus-group sessions, we identified local foods to use as stool-consistency analogs to standardize our measurement in our study population. We used the Quechua versions of the following analogs to measure stool consistency: liquid (water, *api*), semi-liquid (*arrope*), intermediate (milk rice), semi-solid (mashed potatoes), and solid (sausage) (Table 1).

### Analysis of data

#### Descriptive and exploratory

**Fig. 1.**
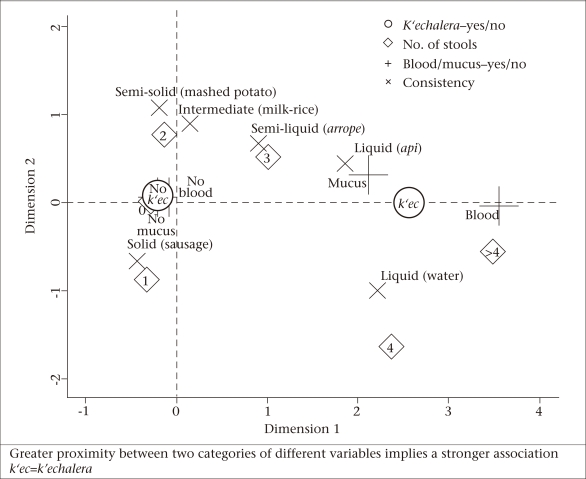
Distribution of modalities of diarrhoeal symptoms of the questionnaire and the reports of *k'echalera* in a scatter plot of the first two factorial axis of a multiple correspondence analysi

The distribution of diarrhoeal symptoms was compared between days with and without reported *k'echalera*. Multiple correspondence analysis (MCA) on the Burt matrix (12) was used for analyzing correspondence among answers to the questionnaire concerning the number of stools, consistency of stool, and presence of blood and mucus. Associations between categories of different variables were simultaneously visualized by a scatter plot of the first two factorial axes (Fig. 1). Symptoms located in close proximity on the plot were interpreted qualitatively to be more highly associated with one another.

#### Estimating sensitivity and specificity

The standard symptoms-based definition (std-diarrhoea) was outlined as the daily passage of at least three watery loose stools or at least one stool containing blood or mucus. Reported *k'echalera* were contrasted with those of std-diarrhoea (Table 2). We assumed that both *k'echalera* and std-diarrhoea were susceptible to diagnostic error. We assumed that symptom reports may be subject to measurement error depending on how attentive the caregiver was to the child's regular defaecation patterns. In addition, cultural norms when reporting to the field staff may contribute to reporting bias (13). Since the standard methods of calculating diagnostic statistics assume that the ‘gold standard’ method is the truth (an assumption that may not reasonably hold in this analysis), we estimated sensitivity (*Se*) and specificity (*Sp*) using Bayesian methods (14,15), which allow both metrics—*k'echalera* and std-diarrhoea—to be measured with error.

**Fig. 2.**
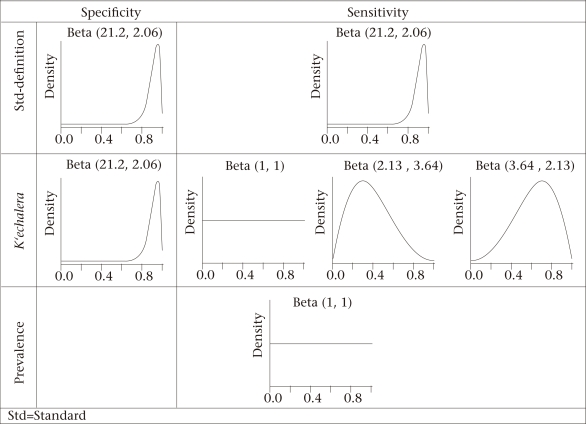
Prior distributions for sensitivity and specificity of *k'echalera* and for functional definition of diarrhoea based on reported symptoms

The beta probability distribution was used for modelling prior beliefs (15). Informative priors (greater certitude) for the sensitivity and specificity (*^d^Se* and *^d^Sp*) of std-diarrhoea were adopted. We assumed std-diarrhoea to be highly sensitive and specific, i.e. a beta distribution with mode=0.95 and 95% chances of being at least 0.8 (Fig. 2, upper-row panels). Given the high observed specificity (Table 2) and the negative predictive value of *k'echalera*, informative (beta) priors were used for the sensitivity and specificity (*^k^Se* and *^k^Sp*) of *k'echalera*. We assumed *^k^Sp* to have a mode=0.95 but 95% chances of being at least 0.80. More uncertainty was assumed about the knowledge of *^k^Se*, and the following three priors were assessed:

- Full uncertainty (uninformative prior: *^k^Se* ~ beta (1,1));
- Vague optimistic prior (mode=0.7 and 95% chances of being at least 0.3); and
- Vague pessimistic prior (mode=0.3 and 95% chances of being at most 0.70).

Finally, a prior assuming complete ignorance of the prevalence of diarrhoea (λ) was also evaluated (λ ~ beta (1,1)). Figure 2 displays the assumed prior uncertainty on *^d^Se*, *^d^Sp*, *^k^Se,* and *^k^Sp*.

## RESULTS

### 

The distribution of the diarrhoeal symptoms is reported in Table 1 for days with and without *k'echalera* from the pre-intervention study and days with *k'echalera* from the post-intervention follow-up. A day without *k'echalera* was characterized by a median of one stool, mostly solid or semi-solid (69.8%). Although in a much lower proportion, blood and mucus were also reported in days without *k'echalera*. Days with *k'echalera* in the pre-intervention study were characterized by a median of three stools during the last 24 hours, a predominant proportion of watery stool (81.1%), and higher frequency of blood or presence of mucus compared to days without *k'echalera*. Watery stool was defined as one that would take the shape of the container (16,17). Among the categories of watery loose stools, ‘milk-rice’ was equally likely in both the days with and without *k'echalera*. Similar patterns were observed in the post-intervention data with a much larger sample-size. Here, the proportion of watery stool was higher (93.2%) than that at baseline (81.1%), owing to the increase of liquid and decrease of solid and semi-solid consistencies. A characterization of days without *k'echalera* was not provided for the post-intervention period because data on diarrhoeal symptoms were collected only if *k'echalera* was reported.

**Table 1.** Distribution of diarrhoeal symptoms for days with and without *k'echalera* at baseline and in a post-intervention study

Symptom	Pre-intervention (819 children)		Post-intervention (725 children)
	Days without *k'echalera*	Days with *k'echalera*	Days with *k'echalera*
	(n=4,071*)	(n=281*)	(n=4,412*)
No. of stools in the last 24 hours: median (Q1; Q3)	1 (1; 2)	3 (2; 3)	3 (2; 4)
Stool consistency: no.* (%)			
Liquid (water)	142 (3.5)	102 (36.3)	2021 (45.8)
Liquid (*api*†)	76 (1.9)	48 (17.8)	931 (21.1)
Semi-liquid (*arrope*‡)	186 (4.6)	62 (22.1)	912 (20.7)
Intermediate (milk-rice)	177 (4.4)	14 (4.9)	249 (5.6)
Watery stool: total	581 (14.3)	228 (81.1)	4113 (93.2)
Semi-solid (mashed potatoes)	865 (21.3)	24 (8.5)	102 (2.3)
Solid (sausage)	1975 (48.5)	16 (5.7)	6 (0.14)
Solid or semi-solid: total	2840 (69.8)	40 (14.2)	108 (2.5)
Other	1 (0.02)	1 (0.4)	78 (1.8)
Do not know	649 (15.9)	12 (4.3)	113 (2.6)
Blood in stool: no.* (%)	51 (1.25)	39 (13.9)	666 (15.1)
Mucus in stool: no.* (%)	231 (5.7)	97 (34.5)	1965 (44.5)

Pre-intervention data represent once-a-week data. Post-intervention data represent daily data (symptom data collected only when *k'echalera* was reported).

* Number of person-days of observations;

†*Api*: a non-alcoholic thick corn-drink;

‡*Arrope*: a non-alcoholic beverage, a quite thick sweet syrup, produced by adding water to *Prosopis flour* (*borra*)

Figure 1 displays the distribution of categories of the four diarrhoeal symptoms and the *k'echalera* status in a factorial space obtained by MCA. The figure reflects joint symptoms reported for children on the same day of observation. The *k'echalera* contrasts with no *k'echalera* by being at the centre of the categories that characterize diarrhoea, i.e. blood and mucus, the two forms of liquid consistency assessed, and a high number of stools. This suggests that whenever *k'echalera* was reported, the diarrhoeal symptoms were reported too. Conversely, no *k'echalera* was reported in the absence of blood, mucus, solid or semi-solid stools. Interestingly, three stools per day and semi-liquid stool-consistency modalities fall approximately equidistant between *k'echalera* and no *k'echalera* classifications. This suggests that these symptom-categories prevail where the two classifications begin to overlap. Indeed, from all the semi-liquid reports in days with *k'echalera* (n=61), 85.5% were given when ≥2 stools were reported (35.5% correspond to 2 stools). Conversely, 95.2% (n=183) of the semi-liquid stools in *k'echalera*-free days were reported when ≤3 stools were reported [14.0%, 34.9%, and 40.3% for 3, 2 and 1 stool(s) respectively]. The intermediate milk-rice and semi-solid stool consistencies fall closer to days without *k'echalera* because both of these were frequently reported together with two stools.

#### Observed sensitivity and specificity

Table 2 shows the distribution of the days with *k'echalera* across the combination of diarrhoeal symptoms that make the standard definition—std-diarrhoea. The pre-intervention data were used because diarrhoeal symptoms were available for both the days with and without *k'echalera*. Assuming that std-diarrhoea is the gold standard, the observed sensitivity of *k'echalera* was 36% (177/492). The main reason for a low sensitivity was the large number of false negatives. From the 315 days without *k'echalera* but positive according to std-diarrhoea, 104 reported at least three watery loose stools, 16 reported at least one stool with blood, 168 reported mucus, and 26 reported both mucus and blood (Table 3). The reasons for the 100 apparent false positives are presented in Table 3. The prevalence calculated following the std-diarrhoea definition yielded 12.2% (492/4,026) while a prevalence following the *k'echalera* definition suggested 6.9% (277/4,026).

**Table 2.** Reported *k'echalera* compared to standard symptoms-based definition of diarrhoea (pre-intervention data)

	Std-diarrhoea	
*K'echalera*	Days with	Days without
Days with	177	100
Days without	315	3,434

The observed specificity of 97.2% (3,434/3,534) and the negative predictive value of 91.2% (3,434/3,749) were high.

#### Modelling sensitivity and specificity

Assuming that both *k'echalera* and std-diarrhoea are subject to diagnostic error or recall bias, the sensitivity and specificity estimates using the uncertainty levels, displayed in Figure 2, are presented in Table 4. Note that we presumed to be more certain on the high specificity of *k'echalera* and on the high *Se* and *Sp* of the standard definition.

Regardless of prior beliefs about the sensitivity of *k'echalera* (uninformative, vaguely optimistic, and vaguely pessimistic), *kSe* was always estimated higher than the observed values calculated from Table 2. Introducing a reasonable level of uncertainty in the report of the std-diarrhoea symptoms led to an important increase in *^k^Se* to 50% with the pessimistic prior and 62% with the optimistic one (Table 4). *^k^Sp* was always high. The prevalence of diarrhoeawas estimated to be around 7.7% assuming uninformative and optimistic priors and 9.5% assuming a pessimistic prior for *^k^Se* (Table 4).

**Table 3.** Reasons of false-negative and false-positive reports of *k'echalera* using standard symptoms-based definition of diarrhoea as gold standard

Category	Reported symptom	No.	%
False negatives	≥3 watery loose stools, no blood, no mucus	104	33.1
	≥1 stool with only blood	16	5.1
	≥1 stool with only mucus	168	53.5
	≥1 stool with both blood and mucus	26	8.3
	Missing	1	0.3
	Total	315	
False positives	<3 stools, no blood, no mucus	74	74.0
	3 solid or semi-solid stools, no blood, no mucus	10	10.0
	Missing	16	16.0
	Total	100	

## DISCUSSION

We evaluated the meaning of the vernacular term *k'echalera* as a mother/caregiver's diagnosis of diarrhoea in rural Bolivian settings and compared its reporting with an internationally-standardized, symptom-based definition of diarrhoea. We found that the caregivers used the term *k'echalera* to reflect a noticeable change in the child's regular defaecation patterns characterized by an increase in the frequency of bowel movement and a change in the stool consistency (a median of three watery stools during the last 24 hours–81.1% of the stools in days with *k'echalera* had a watery consistency, and a greater proportion of blood and mucus compared to days without *k'echalera*). The proportion of watery stool was confirmed to be greater (93.2%) in *k'echalera* days when measured in the post-intervention data. We found some divergence in the vernacular use of *k'echalera* and the international standard definition of diarrhoea. A *k'echalera* case was strongly associated with liquid and semi-liquid stools that differ clearly from solid stool. However, the intermediate stool-consistency level (stool looking like milk-rice), which fits into the definition of watery loose stool (16,17), did not help discriminate between *k'echalera* and non-*k'echalera*. Mucus was reported during days without *k'echalera* in a much lower proportion but enough to increase appreciably the number of false positives.

**Table 4.** Estimates of sensitivity and specificity of *k'echalera* and the standard definition allowing for uncertainty in their reporting accuracy (pre-intervention data)

Estimate for	Prior for *k'echalera*	Sensitivity	Specificity
*K'echalera*	Uninformative	60.8 (38.1; 97.4)*	97.5 (96.8; 98.6)
	Optimistic	61.9 (39,3; 91.7)	97.6 (96.8; 98.6)
	Pessimistic	49.6 (36.1; 77.6)	97.6 (96.8; 98.6)
Std-diarrhoea	Uninformative	92.4 (78.2; 98.8)	94.4 (91.4; 98.9)
	Optimistic	92.2 (78.3; 98.8)	94.3 (91.7; 98.6)
	Pessimistic	92.5 (78.4; 98.8)	96.1 (92.7; 99.3)
Prevalence of diarrhoea	Uninformative	7.7 (4.5; 12.8)		
	Optimistic	7.6 (4.8; 12.4)		
	Pessimistic	9.5 (5.8; 13.3)		


*Posterior median (credible interval);

Std=standard

These observed reporting differences led to a low sensitivity of the vernacular term compared to the standard symptoms-based definition of diarrhoea. The reporting differences led principally to false negatives characterized by episodes with high stool frequency and intermediate consistencies, or days with at least one stool with mucus. The specificity and the negative predictive value of *k'echalera* were consistently high. A Bayesian analysis that allowed for measurement error in both *k'echalera* and symptom-based definition of diarrhoea (a scenario that we argue more accurately reflects real measurement conditions) increased the estimated sensitivity of the vernacular term from 36% to 50-62%.

We also hypothesize that the two main sources of measurement error might both account for discrepancies between *k'echalera* and the symptom reports: (a) perception/detection by the caregiver, influenced by how much time the caregiver spends with the child and how much attention she pays to stool symptoms and (b) the caregiver reporting to the field staff, influenced by cultural norms, practices, and social desirability, and the relationship between the caregiver and the field staff. Moreover, we wished to allow for possible deviations of std-diarrhoea from the actual changes in defaecation patterns in the study setting. To allow for this phenomenon, we estimated the sensitivity of the term *k'echalera* using Bayesian techniques that allowed for a reasonable level of uncertainty in the report of symptoms. A higher sensitivity was then obtained and validated through analysis of sensitivity to the priors.

This approach yielded greater *Se* estimates for maternal reports of diarrhoea than were obtained by treating the symptoms-based definition as gold standard (Table 2). Baqui and colleagues actually assumed that the mother's definition is the gold standard (1). Their data suggest that the *Se* of the mother's definition compared to the standard definition is 68% (in line with our 61% estimate using uninformative and vague optimistic priors for *^k^Se*). A study in South Africa reported even a higher sensitivity of 89% for the mothers’ report (18). However, the latter estimate was obtained comparing the occurrence of diarrhoea over the 1 to 2-month recall period with the occurrence of symptoms in the same period. In contrast, our study, like others (1), compared the reports of symptoms and occurrence of *k'echalera* corresponding to one day of observation. Thomas *et al.* provided the *Se* and *Sp* estimates for mothers’ reports of diarrhoea being 79% and 94% respectively (19). A study in the Cebu Island of the Philippines provided the *Se* and *Sp* estimates of maternal symptom-based diagnosis and compared with physicians’ diagnosis (20). The diagnosis of diarrhoea had a sensitivity of 95-97% and a specificity of 80% when based on the maternal reports of frequent passing of liquid stools. This suggests that mothers were able to retrospectively report the signs and symptoms of their children accurately for interview-based diagnosis. Those *Se* and *Sp* concur withour assumption on the priors for the symptoms-based definition in the Bayesian analysis.

Our crude prevalence estimates fall between 6.9% (*k'echalera*) and 12.2% (symptoms-based diarrhoea). This suggests that, in our study setting, mothersdo not identify diarrhoea very consistently with the international definition. In contrast to other cultures, in many cases, mothers reported the presence of mucus and milk-rice stool consistency as ‘normal’ whereas elsewhere this would be reported as diarrhoea, e.g. Bangladesh (1), South Africa (18), and Kenya (19). We found a high prevalence of malnutrition, especially among wasted children (data not shown). This health status was often accompanied with malabsorption of food and chronic diarrhoea with milk-rice stool consistency. The malabsorption of food and the resulting unshaped stool, which is often accompanied with mucus, is a well-described physiological phenomenon (4). We presume that such health status was perceived as normal by the mother and reported as a day without *k'echalera*.

We believe that the prevalence of diarrhoea lies between that of the *k'echalera* and std-diarrhoea estimates and that the uncertainty assumed during the Bayesian analysis is a reasonable approximation (7.6-9.5%). The disadvantage of this approach is that good care should be taken when choosing the priors, since the final estimates may be sensitive to their choice.

### Conclusions

In the rural Bolivian population, the term *k'echalera* was used for reporting a true change in the defaecation patterns of children aged less than five years; *k'echalera* was strongly associated with the symptoms that are used in the symptoms-based standard definition. However, the intermediate (milk rice) stool consistency and presence of mucus, part of the standard definition, were frequently reported in days without *k'echalera* and were responsible for numerous false-negative results. The use of *k'echalera* would, thus, identify fewer cases of diarrhoea than a symptom-based definition in rural Bolivia. We estimated an average sensitivity of *k'echalera* of at least 50% and a specificity of 97% when allowing for uncertainty on both *k'echalera* and report of symptoms. The low sensitivity of *k'echalera* compared to the standard definition may be due, in part, to caregivers perceiving as normal chronic, low-level diarrhoeal symptoms that classify children as diarrhoeic in other settings. Additional studies that report the relationship between vernacular and symptoms-based definition of diarrhoea in other populations will help investigators judge the comparability of results from field studies conducted in different cultural contexts.

## ACKNOWLEDGEMENTS

The BoliviaWET trial was funded by the National Institutes of Health (Award No. R01AI50087-01IH). Gonzalo Durán Pacheco also received a stipend from the Stipendiumkommission of the Amt für Ausbildungsbeiträge of the Canton of Basel, Switzerland. The authors thank the participating families and acknowledge the support of the study communities in Bolivia. They also acknowledge the field staff of the BoliviaWET study for their relentless commitment during data-collection. The authors specifically thank Stefan Indengard and Michael Hobbins for their valuable contributions in assessing the vernacular terminology of childhood diarrhoea in rural Bolivia. They are grateful to Ada Armaza for her valuable comments on a previous version of the manuscript.
